# Supplementary material for: Patterns of Intron Gain and Loss in Fungi
Source: PLoS Biol. 2004 Nov 30;2(12):e422. doi: 10.1371/journal.pbio.0020422 (PMC532390; doi:10.1371/journal.pbio.0020422)
Supplement: Table S1 — Also available at http://genes.mit.edu/NielsenEtAl/. (4.3 MB ZIP). [file pbio.0020422.st001.zip › NielsenEtAl/html/60.html]

AN3017.1.NCU08783.1.MG03387.1.FG01765.1


```
 CLUSTAL W (1.82) Multiple Sequence Alignments - Introns Inserted


Sequence 1: AN3017.1	416 aa
Sequence 2: FG01765.1	399 aa
Sequence 3: MG03387.1	422 aa
Sequence 4: NCU08783.1	430 aa
Alignment Length: 453 aa
Number Identitical Residues: 97 aa
Alignment Score (without introns) 6435


MG03387.1 	--MDSKSFGKLPETANTASVQPYSIQIPKHDVAQMQQLVKMSPIAS-ACYENSLPNGDNS
NCU08783.1	MGSVSPTFSTLPAGV-KVQPTPFRINVAESDLAQFRSLIQQAIIPPEQFYNQHANAATGK
FG01765.1 	---MADSYAKIPSGA-LVQPTPFRVSIEDERVEELKLLVKLGKIAN-PTYESTQK--EHN
AN3017.1  	---MTAPFTKLPSTA-TITPSSFRVAIPDEQLSDLKTLLRLSRTAP-QTYENLQE--DGR
          	    : .: .:*  .      .: : : .  : ::: *:: .  .    *:.        

MG03387.1 	YGLGRDWLVAAKERWANSFDW2NKTEARLNGYNHFIAKVADEQLGQTFDIHFVALFSQAR
NCU08783.1	FGITRDWLIQARDYWLNTYDW~RAQETFINSFPQYKQTVVGPTSGQTFDLHFAALFSLRK
FG01765.1 	YGITHQWLTDAKDAWMK-FDW2RAAEKRINSFNHWKVPVHDTKG--DFDMHFTGLFSTKP
AN3017.1  	YGVTHKWLSTMKEEWLNNFDW2RAVEEHANIFPQYTTEIEG------LTIHFAALFSEKA
          	:*: :.**   :: * :.:** .  *   * : ::   : .      : :**..***   

MG03387.1 	QPVKPIILLHGWPG~SFLEFLSMLDLLKDKYSPEDLPYHIVVPSLPGYLFSSAP--PLDR
NCU08783.1	D-AIPIIFMHGWPG~SFLEFVPMLDILRSRYTPETLPYHVIVPSIPDYGFSSRPNDSALQ
FG01765.1 	N-AIPIVMVHGWPG~SFLEYLGVLSILKDRYTSETLPYHIIIPSLPGFAFSSKP--PMER
AN3017.1  	D-AVPIVLLHGWPG1SYFEFLPLLQLFKDEYSPSTLPYHLIVPSLPGYTFSSGP--PLDR
          	: . **:::***** *::*:: :*.:::..*:.. ****:::**:*.: *** *  .  :

MG03387.1 	DFGLRDVARLMDSLMVEHLGFGESGYIAQGGDVGSRVCRVLAAKYDRCLG~TLL~NYNRI
NCU08783.1	ELNMEFAAEAMNELMLS-LGFGSTGYVAQGGDVGYALARAMANNHDACKT~SHL~NMFMF
FG01765.1 	DFCIEDASRIINTLMVQ-LGFG-SGYVIQGGDLGSIVACELATNYKECKA1LHL~NMC-M
AN3017.1  	DFKNADSARIVDKLMRG-LGFS-GGYVSQGGDIGSTVARILAVGYNSCKA1VHL1NFCPL
          	::     :. :: **   ***.  **: ****:*  :.  :*  :. *     * *   :

MG03387.1 	GKPEGSAG-PEALSEEEKAGLERCKWFDSVGTAYAMAHATRPSTMGLVLSSSPIALLAW~
NCU08783.1	TPDQFAACQQEPLTEREEKLLTGTNAWIKQGSAYAYEHGTRPSTIALTLSTNPVAMLAW~
FG01765.1 	VPEPSTVT-GEVT-EAEKQALERGKDFFTRGSAYAFTHGTKPSTIGLVLSTSPLALLTW~
AN3017.1  	VKRPEGIS-DEYHNELEKRGFERSNWFLTYGKAYAEEHGTKPATIGHVLASSPLALLAW2
          	          *  .* *:  :   : : . *.***  *.*:*:*:. .*::.*:*:*:* 

MG03387.1 	VGEKFVDWSDPKSYPPEEGTGYSTDLMDEVLLSASLYWLTGTPPRCLYSYRE~TYDVGSG
NCU08783.1	MGEKFIEWSDNRKHG-----GSRPLSLDTILDGVSLYWFSGCFPRTMWSYRS~LVPAIGA
FG01765.1 	IGEKFRDWTDIEPP------------IDETLTSVSLYWLTDTYPTSIYAYRH~M-PVFGG
AN3017.1  	IGEKYLDWPDTPFS------------KHKILELVTLYWFTESFPRAIYPYRQ0TNPIPPG
          	:***: :*.*                 .  *  .:***::   *  ::.**        .

MG03387.1 	KKKWHELPDYHIRAPKKFGFTWFPLDLAPIPKSWIETTG--DLVWFRRHEV~GGHFAAME
NCU08783.1	TA--VVQESLSVQKP--FGYSAFPVEIGTLPRTWGKKLFGDRLAYYKEHEV~GGHFAALQ
FG01765.1 	P---PGKPMPYITKP--MSYSWLPKEVAPKPVAWVSSVG--NLVHYKRHEG~GGHFAAFE
AN3017.1  	PS--PFLNELYIHKP--FGFSYFPKELIPVPESWVAKTG--NLVYFKQHSE0GGHFAALE
          	           :  *  :.:: :* :: . * :*  .     *. ::.*.  ******::

MG03387.1 	QPVALLGDVEEFCKLFG-----~------------------
NCU08783.1	EPENFLDDIEEFVRFIAPKVGL~AKGGKSA-----------
FG01765.1 	RPGELLGAVEEFVKQISADGSL~KI----------------
AN3017.1  	LPEKFKEDLSEFVTQVWSSASK2TYLALRYRSPPTAKTAQK
          	 *  :   :.**   . .. .     .    :..::.::..
```
